# Myeloid sarcoma of the pancreas: A case report and literature review

**DOI:** 10.1097/MD.0000000000024913

**Published:** 2021-04-02

**Authors:** Kangze Wu, Xuzhao Zhang, Bo Zhang

**Affiliations:** a Department of Surgery; b Department of Hematology, Second Affiliated Hospital, School of Medicine, Zhejiang University.

**Keywords:** acute myeloid leukemia, epigastralgia, myeloid sarcoma, pancreatic cancer

## Abstract

**Introduction:**

Myeloid sarcoma (MS) is an extramedullary mass, consisting of myeloid blasts with or without maturation, which efface the normal tissue architecture. It occurs mainly in lymph nodes, skin and soft tissue, testis, bone, peritoneum, and gastrointestinal tract, but rarely in the pancreas. Because their clinical courses, treatments, and prognoses are quite different, it is crucially important to distinguish between MS and pancreatic cancer.

**Patient concerns:**

We herein report a rare case of acute myeloid leukemia (AML) which presented with a pancreatic mass that mimicked pancreatic cancer.

Diagnosis: The diagnosis of MS was established based on immunohistochemical (IHC) analysis and bone marrow examination which revealed neoplastic cells with CD34+/CD117+.

**Interventions:**

The patient was actively treated with chemotherapy.

**Outcomes:**

After 4 cycles of chemotherapy, the lesion in pancreas was significantly reduced, and the patient is still receiving further chemotherapy.

**Conclusion:**

When we encounter a patient suspected of pancreatic cancer with blood cell abnormalities and no significant increase in carbohydrate antigen 19-9 (CA19-9), we need to be aware of the possibility of pancreatic MS. Preoperative pathological biopsy and IHC are indispensable. Misdiagnosis is common if we rely solely on imaging.

## 1 Introduction

Acute myeloid leukemia (AML) consists of a group of relatively well-defined hematopoietic neoplasms involving precursor cells committed to the myeloid line of cellular development. AML is the most common acute leukemia (AL) in adults, accounting for about 80% of adult AL,^[1,2]^ while it accounts for less than 10% of AL in children under the age of 10. According to the report, the incidence rate in the United States and Europe is 3 to 5 cases per 100,000 people.^[3–5]^ Myeloid sarcoma (MS) is a distinct clinical presentation of any subtype of AML.^[6]^ The clinical manifestations of MS vary according to the size and location of the mass. When it invades the pancreas, its clinical manifestation may be similar to that of pancreatic cancer.

We herein report a rare case of AML incidentally which presented with a pancreatic mass that mimicked pancreatic cancer.

## 2 Case report

A 32-year-old woman was admitted to our department because of a sudden epigastric pain. She denied any other symptoms, including nausea, vomiting, diarrhea, and weight loss. She had no significant family history.

Basic laboratory tests were performed during her hospital admission and revealed that her white blood cell count was lower than normal (2.2 × 10^9^/L, reference range >4 × 10^9^/L). The elevated total bilirubin (44.0 μmol/L, reference range <17.1 μmol/L) and direct bilirubin (14.2 μmol/L, reference range <6.8 μmol/L) were also noted, which was strongly suggestive of obstructive jaundice. The carbohydrate antigen (CA) 19-9 increased slightly (54.5 U/ml, reference range <37.0 U/ml). Throughout physical examination, no abnormal findings were revealed except for jaundice and positive Murphy syndrome.

**Figure 1:**
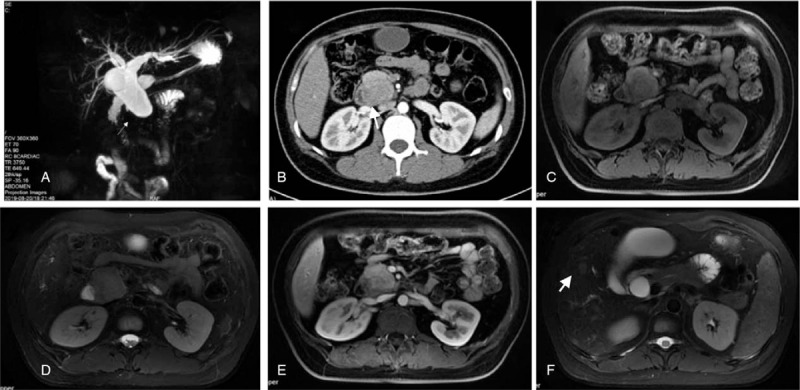
A: Magnetic resonance cholangiopancreatography showed dilated intrahepatic and extrahepatic bile ducts, enlarged gallbladder and tumor mass (white arrow); B: CT image of tumor (white arrow); C (T1 image) and D (T2 image): The mass abnormal signal of the pancreatic head of the lower common bile duct was about 44 mm × 41 mm, showing slightly longer T1 and longer T2 signal; E: The enhanced enhancement was slightly weaker than that of the surrounding pancreatic tissue; F: Liver lesion (white arrow).

**Figure 2:**
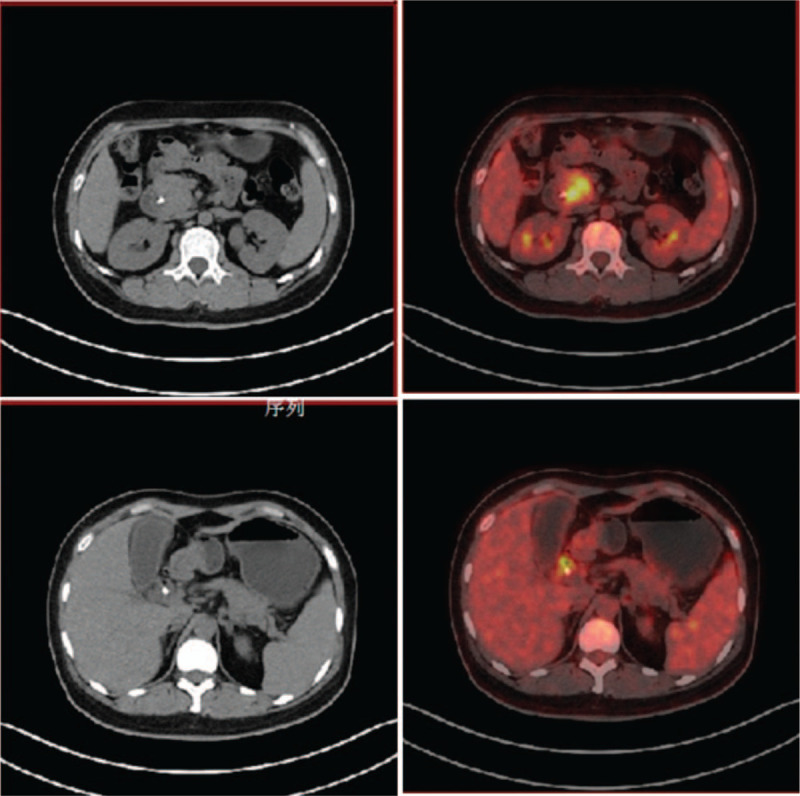
Soft tissue mass was seen in the head of the pancreas, the size was about 4.11 cm/3.79 cm, and the radioactivity uptake was increased. Interestingly, on PET-CT scan, liver lesions were invisible.

**Figure 3:**
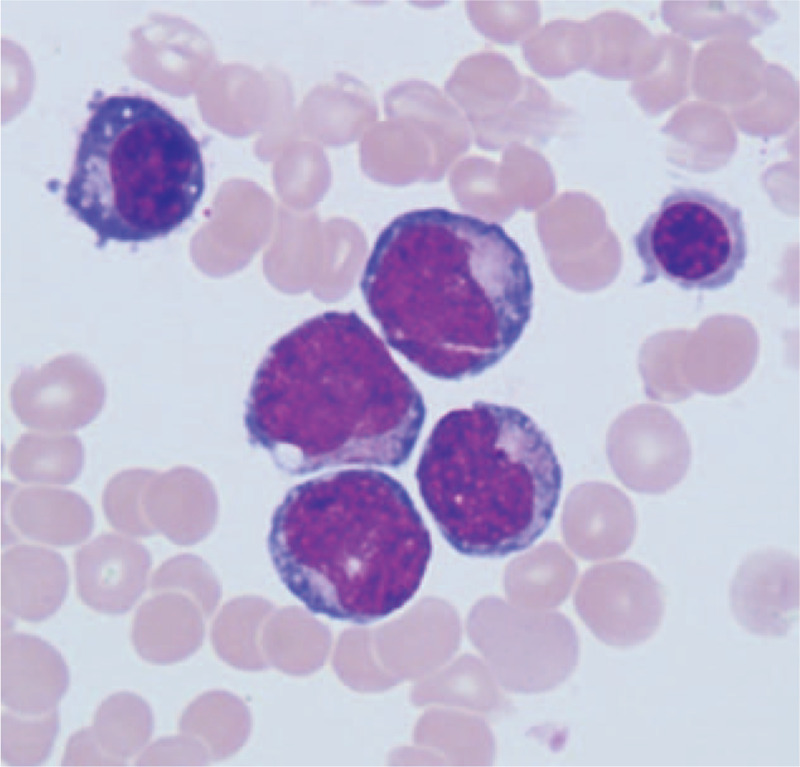
Morphology of bone marrow under bone marrow microscope. Typical malignant cells can be seen around red blood cells. The malignant cells are large with abundant cytoplasm and large nuclei.

**Figure 4:**
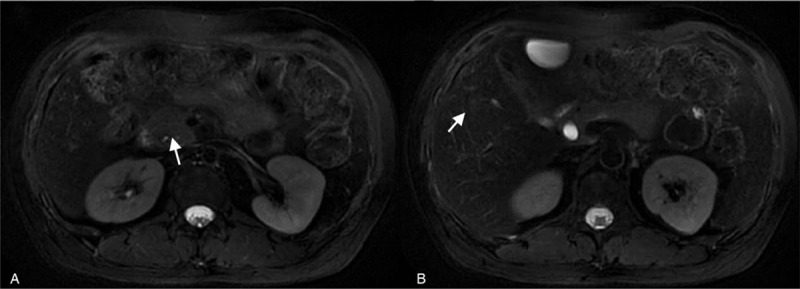
A: After 2 cycles of chemotherapy, MRI showed that the lesions in the uncinate process of the head of the pancreas were significantly reduced (white arrow); B: The focus of the liver has also become blurred. (white arrow).

Abdominal enhanced computed tomography (CT) revealed abnormalities suggestive of malignancy in the pancreatic head and multiple mild enhancement foci in the liver, which may be metastases (Fig. 1). Magnetic resonance imaging (MRI) also showed a possibly malignant mass in the uncinate process of the head of the pancreas, causing intra-and-extra hepatic duct dilation (Fig. 1). Positron emission tomography CT (PET-CT) revealed pathologic lesions in the head of the pancreas with increased glucose metabolism, which may be neoplastic lesions, while the glucose metabolism of intrahepatic lesions did not increase significantly (Fig. 2). An endoscopic ultrasound-guided fine-needle aspiration biopsy was performed in pancreas, but the results revealed that a small amount of mucosal epithelium could be seen and no evidence of malignancy was found. An ultrasound-guided percutaneous biopsy of the liver was also performed, and results revealed MS. Immunohistochemical analysis revealed neoplastic cells that were positive for CD4, CD10, CD68, ki-67, Bcl-2, MPO, and CD34. Therefore, bone marrow examination was performed and results revealed neoplastic cells were CD34+/CD117+, which was highly suggestive of an AML (Fig. 3). After that, the patient had a genetic test, and the results showed that the patient was positive for AML1/ETO fusion gene. Thus, a diagnosis of AML was made. The patient was then started on an IA chemotherapy regimen (idarubicin and cytarabine). The patient was given 15 mg idarubicin in the first day and 10 mg in the next 2 days. A hundred mg cytarabine are used every 12 hours from day 1 to day 7. After 4 cycles of chemotherapy, the lesion in pancreas was significantly reduced (Fig. 4).

The patient is currently followed up every 2 months. The chemotherapy is evaluated as partial response, which means the sum of the maximum diameter of the target lesion is reduced by more than or equal to 30% for at least 4 weeks. The follow-up treatment is still in progress.

## 3 Discussion

Haematological malignancies sometimes may manifest as a soft mass, such as MS, which often cause misdiagnosis. The incidence of MS in AML is not high, only 2.5% to 9.1%.^[7–9]^ This rare disease can occur in any part of the body, and the clinical manifestations vary depending on where it occurs. According to the existing literature, MS occurs mainly in lymph nodes, skin and soft tissue, testis, bone, peritoneum, and gastrointestinal tract.^[8,10,11]^ Therefore, the clinical understanding of the disease is relatively limited, which is prone to misdiagnosis and delayed treatment.^[7,8]^

We searched for cases of pancreatic MS and found that the age of pancreatic MS patients ranges from 19 to 64 years old, and most of them are older than 30 years old, while there is only one 19-year-old patient. Pancreatic MS seems to be more likely to occur in adults.^[12–20]^

In terms of symptoms, we found that almost all reported pancreatic MS had symptoms of epigastric pain. Other symptoms, such as jaundice, anemia, weight loss, fatigue, etc, are relatively rare, which may be due to the rarity of related cases (Table 1).^[12–20]^

**Table 1:** Previously reported cases of pancreatic MS.

NO.	Gender/Age	Symptoms	Location	CT	Laboratory examination	Bone marrow	Therapy	response	Reference
1	F/57	General weakness, epigastric pain, jaundice	Head of pancreas	Hypodense mass	Hyperleukocyte	Normal	Whipple procedure and chemotherapy	Unknown	12
2	F/42	Epigastric pain and fatigue	Tail of pancreas	Homogeneous mass	Moderate anemia, thrombocytopenia	15% blasts with Auer rods and trilineage dysplastic features	Chemotherapy	CR	13
3	M/19	Epigastric pain	Head of pancreas	Homogeneous mass	Unknown	Unknown	Chemotherapy	Unknown	14
4	F/48	Epigastric pain, anemia, fever	Tail of pancreas	Hypodense mass	Anemia, low platelet count	Normal	Splenectomy, distal pancreatectomy and chemotherapy	Death	15
5	M/31	Epigastric pain	Head of pancreas	Hypodense mass	Normal	6% blasts and the presence of Auer rods	Chemotherapy	CR	16
6	F/61	Epigastric pain	Head of pancreas	Hypodense mass	Hyperleukocyte	Unknown	Chemotherapy	Death	16
7	M/40	Jaundice, weight loss	Head of pancreas	Hypodense mass	Hypoleukocyte	Normal	Whipple surgery and chemotherapy	CR	17
8	M/64	Hematemesis, melanosis, chest discomfort, infection	Head of pancreas	Homogeneous mass	Hyperleukocyte	70% blasts	Chemotherapy	CR	18
9	M/34	Epigastric pain	Head of pancreas	Hypodense mass	Normal	Unknow	Chemotherapy	CR	19
10	M/36	Epigastric pain	Tail of pancreas	Hypodense mass	Normal	Normal	Chemotherapy	Progress	20
11	M/32	Epigastric pain	Head of pancreas	Hypodense mass	Hypoleukocyte	AML M2	Chemotherapy	Progress	our case

CR = complete response, F = female, M = male, CT = computed tomography, AML = acute myeloid leukemia.

Of the 10 cases we searched, 7 patients had pancreatic MS in the head of the pancreas, and 3 patients in the tail of the pancreas.^[12–20]^ Our case is also a patient with MS at the head of the pancreas, so we suspect that pancreatic MS is more likely to occur at the head of the pancreas, but more related cases are needed.

CT images of pancreatic MS often show low density masses, but homogeneous masses are not uncommon, while pancreatic cancer tends to present as a hypodense mass. PET-CT and MRI are also helpful in the diagnosis of pancreatic tumor. However, they do not play a key role in distinguishing MS from pancreatic cancer. On the contrary, sometimes PET-CT may cause misdiagnosis.^[12–20]^

In our case, PET-CT showed a significant increase in the metabolic rate of the head of the pancreas, but no significant increase in the metabolic rate of intrahepatic lesions and bone marrow, which is suggestive of pancreatic cancer. Hence PET-CT diagnosis should be exercised with caution.

It is reported that most of the patients are hyperleukemic, but a considerable number of patients have normal hemogram, and other conditions such as anemia, hypoleukaemia, and thrombocytopenia also exist. ^[12–20]^ Due to the small number of cases, the typical hemogram of these patients is still uncertain.

Bone marrow biopsy can be a diagnostic method, but due to the existence of isolated extramedullary MS, we cannot rule out MS.^[12,16,19,20]^ The final diagnosis usually depends on immunohistochemistry (IHC).

The treatment of MS mainly depends on chemotherapy and radiotherapy. After intensive AML chemotherapy, radiotherapy can be used as consolidation therapy. This has a certain effect on most of the initial MS. If the chemotherapy fails, we can consider hematopoietic cell transplantation.^[21]^

Although no related studies have reported the prognosis of MS because of its rarity, according to the prognosis of AML,^[22]^ its prognosis is still not optimistic.

To sum up, we have presented a rare adult case of extramedullary MS involving pancreatic head. From the presented case and our reviewed studies, it suggests that when we encounter a patient suspected of pancreatic cancer, who has blood cell abnormalities with no significant increase in CA19–9, we need to be aware of the possibility of pancreatic MS. Preoperative pathological biopsy and IHC are indispensable. Relying solely on imaging is prone to misdiagnosis.

## Author contributions

**Conceptualization:** Kangze Wu.

**Data curation:** Kangze Wu, Xuzhao Zhang, Bo Zhang.

**Methodology:** Kangze Wu.

**Writing – original draft:** Kangze Wu.

**Writing – review & editing:** Kangze Wu.
